# Structure design of unidirectional moisture transfer fabric with high wool content based on multiple differential effects

**DOI:** 10.1038/s41598-025-15179-2

**Published:** 2025-08-16

**Authors:** Ying Chen, Ran Wang

**Affiliations:** Material Science and Design College, Beijing Institute of Fashion Technology, Beijing, China

**Keywords:** Unidirectional moisture transfer, Multiple differential effects, High wool content, Sportswear, Double-sided knitted fabric, The surface diffusion rate, Quality of life, Soft materials

## Abstract

The natural functions of wool fiber, such as natural air permeability, dryness of skin contact, have advantages in the development of sports fabric. So far, the development of unidirectional moisture transfer fabric with high content wool is not yet mature. This paper novelly designed a ply yarn formed by different content of wool and nylon yarn and a double-sided knitted fabric with different wool content in the inner and outer layers based on unidirectional moisture transfer principles. The results showed that the difference in wool content between layers had a significant effect on the cumulative one-way moisture index, while small yarn linear density change had no significant effect on this index, but had a significant effect on the surface diffusion rate. The cumulative one-way moisture conductivity indexes of samples 1/9, 1/8, 1/6, 1/7 and 1/5 were all above 400, with the rating of 5, indicating excellent one-way moisture conductivity, so the one-way moisture conductivity of the fabric with high wool content is realized. The application of this technology will provide better moisture absorption and perspiration performance and wearing comfort for wool sportswear fabrics.

## Introduction

When people sweat outdoors in the heat, the sweat, if not timely evaporated, will accumulate on the surface of skin and causes the micro-environment between the skin and clothing to become hot and humid. This not only brings discomfort but also increases the likelihood of cardiovascular and respiratory diseases^1^. So efficient wicking functions are highly desired for outer door textiles. The difference in wettability between the two sides of the fabric to spontaneously transfer sweat from the inside to the outside, such as Janus fabrics, the relatively hydrophobic side is designed as inner layer and the outer layer is the relatively hydrophilic side. For example, microfiber polyester coils arranged on a bi-layer knitting fabric, is presented. This configuration allows for unidirectional wicking of sweat away from the skin. This exceptional property is achieved by creating differential capillary effects across a predominant combination of microfiber polyester and ordinary hydrophobic polyester^2^.

Materials capable of directing liquid transport in a transient manner are now available. They were achieved through gradient wettability using methods such as physical and chemical modifications or the combination of specially engineered fibers^3–11^. Such as profiled fiber cross section like Coolmax^®^ yarn, nanofibril channel structure Tencel yarn, or Drirelease^®^ yarn with hydrophilic/hydrophobic blended yarn (10–15% cotton + 85–90% polyester)^12^. TransDry^®^ technology is selectively applies a durable water-repellent (DWR) finish to specific cotton yarns within a 100% cotton fabric on the inner side; Wicking Windows™ technology applies a water-repellent finish to the fabric surface in a specific patterned design (e.g., dots, grids), creating distinct hydrophobic and hydrophilic (“windows”) areas; 3XDRY^®^ technology is also a dual-action textile finish. All of these finishes create localized hydrophobic zones, reducing cling and enhancing dryness perception^12–15^.

Kang et al.^4^ added a hydrophilic PAN fiber membrane on top of the cotton layer to form a trilayer progressive wetting structure, the hydrophobic surface is polyester layer. As expected, the progressive wetting behavior was consistent with the previous theoretical model and the efficiency of unidirectional moisture transmission was higher. Inspired by the shorebird’s beak with a conical-shaped liquid pathway for transporting liquid drops, a novel biomimetic porous fabric is proposed by using natural cotton fibers. The resultant biomimetic pore fabric exhibits good water absorption rate of 350%, an outstanding water vapor transmission (> 10000 g/m^2^ /24 h) and high recyclability^7^. In addition, the thermal and moisture comfort of the fiber can also be improved by using the existing fiber for blending component change. Milkweed/polyester plated knitted fabrics for next-to-skin end uses were analyzed by changing the inner and outer layers of plated fabrics and with different polyester/ milkweed blend proportion^11^.

Experts have conducted extensive research and enhancement on fabrics made from moisture-absorbent natural fibers or synthetic fibers. The moisture management performance of silk, wool, and bamboo fabrics has been examined, revealing that fabrics composed of silk or wool fibers can maintain skin dryness even under sweating conditions^16^. keeping the skin surface dry while accelerating the evaporation of sweat to remove heat from the skin surface. Wool sportswear combines the softness and comfort of wool fiber with the functionality required by sportswear, and is suitable for outdoor sports wear^17,18^. Wool fiber in the air layer can effectively block the heat dissipation, good warmth effect, in the cold outdoor sports can keep warm. Wool fibers absorb and wick sweat away, keeping the skin dry, such as the Erdos 1980 base layer compression suit, which uses a honeycomb weave to enhance wicking in areas prone to perspiration. Wool has a soft texture, no foreign body feeling on the skin, and no discomfort caused by clothing rubbing against the skin when exercising. Its fiber structure has certain pores, the air can be freely shuttled, so that the skin can “Breathe”, reduce the muggy feeling and discomfort.

Except materials, Structure of fabrics is also important^19,20^. For example, unlike the direct absorption that occurs in single-layer textiles, bi-layer knitted fabrics with diverse fibers and coil stitches can easily adapt to manage sweat and prevent reverse osmosis^21^. One-way moisture transfer fabric can be achieved through the yarn and structure design of double-layer fabric. The inner layer of the fabric is made of hydrophobic fibers with high linear density, and the outer layer is made of finer hydrophilic fibers. In the middle, yarns with certain moisture absorption and transmission properties can be used to connect the two sides of the fabric to achieve the effect of wicking, such as polyester cotton blended yarn, also can use hydrophobic wet yarn such as polypropylene filament to play a better role in water conduction. This material and structure make use of differential capillary effect and wicking effect. There is differential capillary effect, liquid water conduction and wicking effect between inner and outer layers of the fabric, compared with other double-layer fabric structure models, it has better moisture transfer and fast drying effect.

So far, the development of unidirectional moisture transfer fabric for high content wool and even cashmere is not yet mature. Therefore, this project will use the principle of unidirectional moisture transfer fabric development to design wool/nylon twisted yarns with different wool content and different fineness, and use double-layer knitted fabric structure design to realize unidirectional moisture transfer function, and its influencing factors were statistically analyzed. The application of this technology will provide better moisture absorption and perspiration performance and wearing comfort for wool sportswear fabrics.

## Materials and methods

### Yarn structure design

**Fig. 1:**
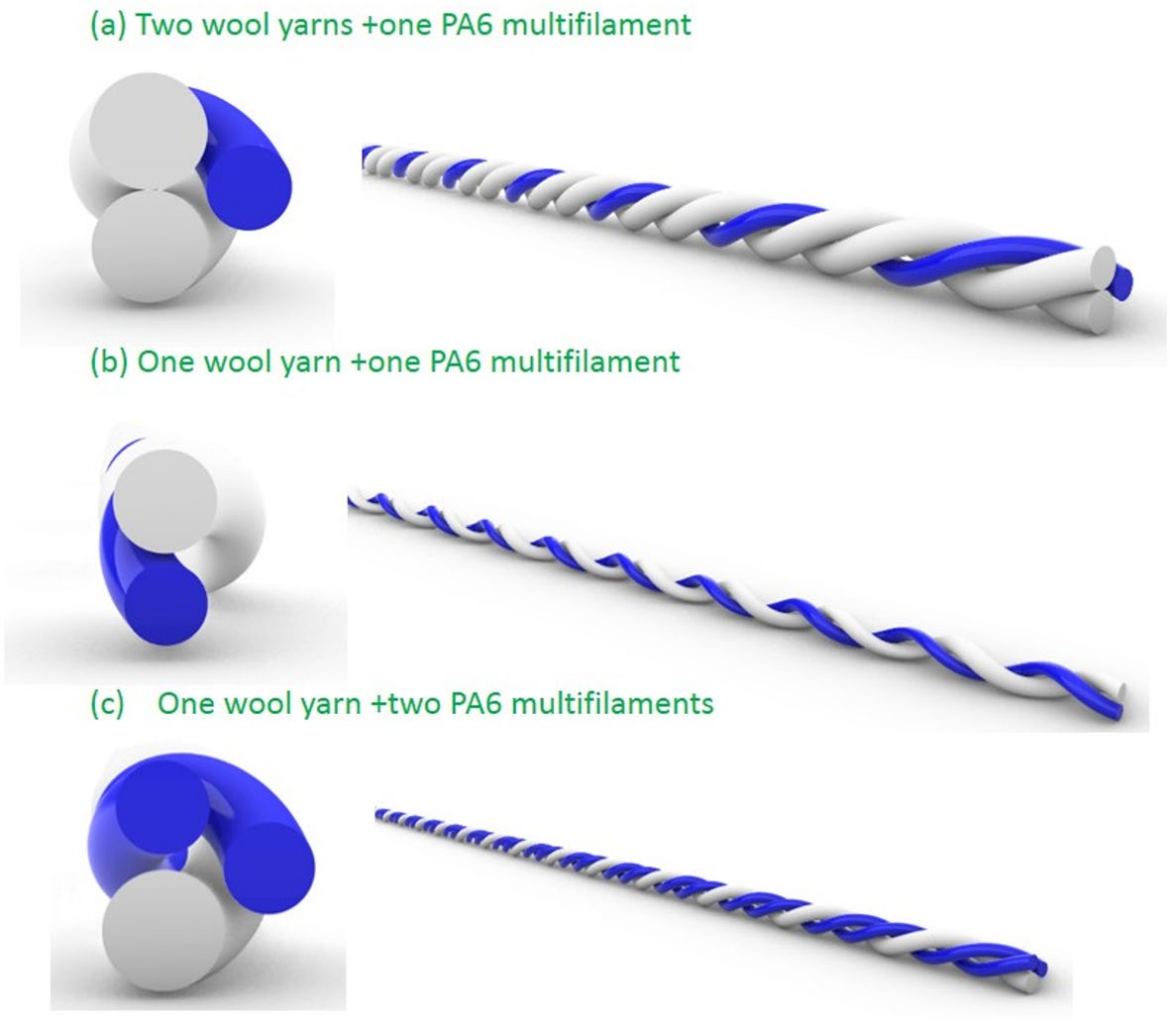
schematic diagram of yarn structure.

The raw materials are 80-count yarns spun from wool fibers with a fineness of 20 μm (Zhangjiagang Yangtse Spinning Ltd.) and nylon multifilament with different fineness (China Resources Yantai Nylon Co., Ltd.). the twist of wool yarn is 756 twist/m and the twist of both doubled and tripled yarns is 385.7 twist/m. The structural parameters of the multifilament were shown in Table 1. Different numbers of wool yarns or nylon multifilament (1 or 2) were combined and twisted to form double or triple strands, as shown in Fig. 1.

**Table 1:** Structure of combined twist ply yarn.

Combined twist ply yarn	Metric count of wool yarn	Number of wool yarn	Nylon multifilament	Number of Nylon multifilament
1^#^	80	2	20D/24f	1
2^#^	80	2	40D/36f	1
3^#^	80	2	70D/68f	1
4^#^	80	1	20D/24f	1
5^#^	80	1	40D/36f	1
6^#^	80	1	70D/68f	1
7^#^	80	1	20D/24f	2
8^#^	80	1	40D/36f	2
9^#^	80	1	70D/68f	2

### Fabric structure design

The above strands are combined to form a double-sided fabric, and the combination method was shown in Table 2. The inner fabric was knitted with yarn with smaller wool content and finer fineness, and the outer layer is woven with yarn with more wool content and coarser fineness, forming a double-layer differential effect of hygroscopicity and fineness to achieve one-way moisture transfer. Table 2 showed the different combinations of inner and outer yarns. The larger the number, the finer the fineness.

**Table 2:** Yarn combination and number of fabric.

Number of fabric	Number of outer- layer yarn	Number of inner- layer yarn	Number of fabric	Number of outer- layer yarn	Number of inner- layer yarn	Number of fabric	Number of outer- layer yarn	Number of inner- layer yarn
1/9	1	9	2/7	2	7	5/9	5	9
1/8	1	8	2/7	2	5	5/8	5	8
1/6	1	6	2/3	2	3	5/6	5	6
1/7	1	7	3/9	3	9	5/7	5	7
1/5	1	5	3/8	3	8	7/9	7	9
1/3	1	3	3/6	3	6	7/8	7	8
1/2	1	2	3/7	3	7	7/6	7	6
2/9	2	9	3/5	3	5	6/9	6	9
2/8	2	8	4/5	4	5	6/8	6	8
2/6	2	6				8/9	8	9

### Knitting

**Fig. 2:**
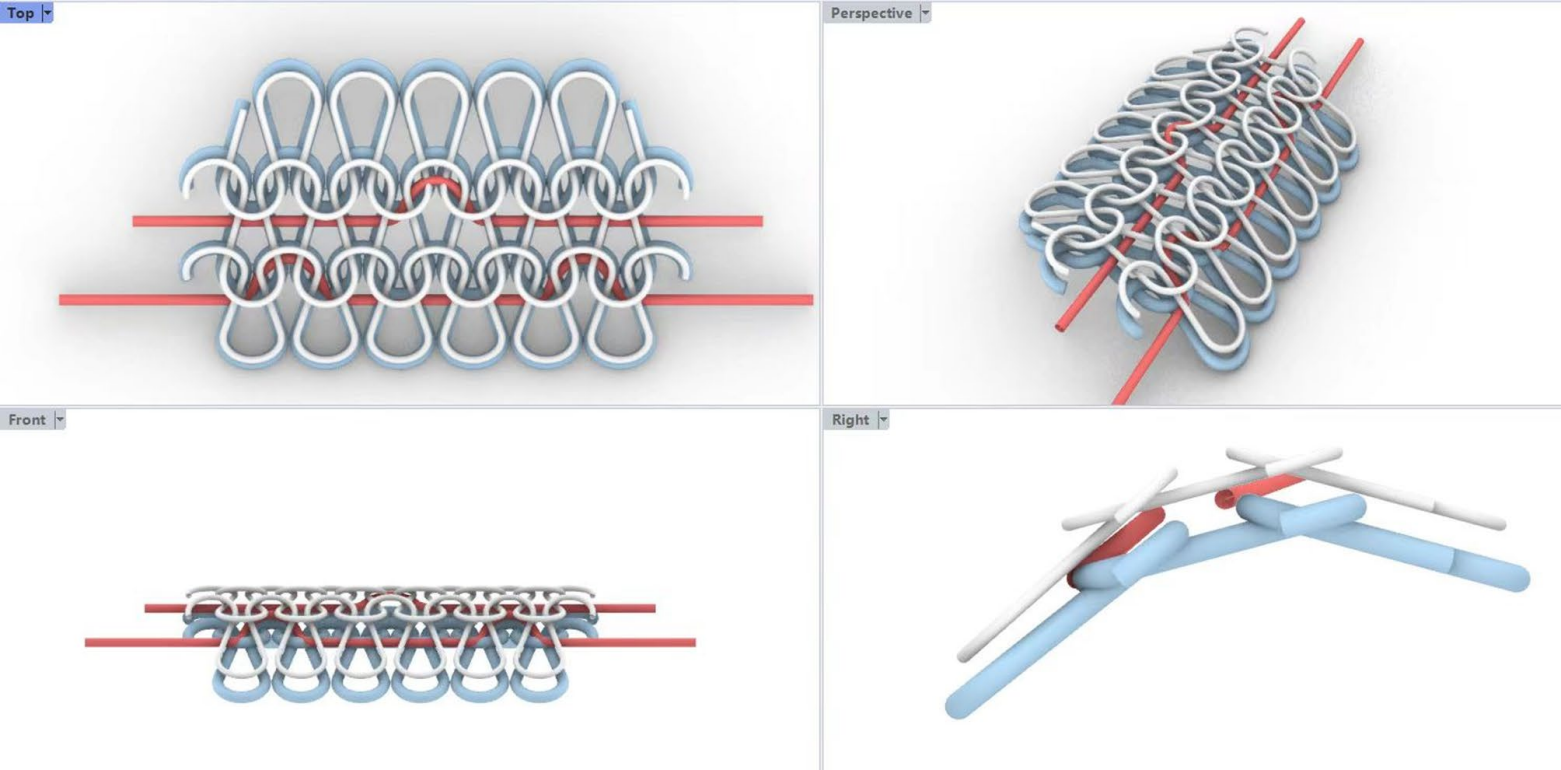
Structure of wool/nylon double-faced fabric.

The above designed fabrics are woven by Biwo 18-needle computerized flat knitting machine. The traction belt spacing of flat knitting machine is 18 needles / inch, the width of the traction belt is 48 inches, and the traction speed is 1.3 m / second. The weaves of the front and back fabrics are weft plain needles, and the fabric density is the same. The structure diagram was shown in Fig. 2. The thicker blue yarn is the outer fabric yarn, and the finer white yarn is the inner fabric yarn. The red yarn is a two-layer knotted yarn, the same as the outer yarn.

### Characteration

#### Contact angle

SDC-200 S contact angle tester was used to test the instantaneous contact angle of inner and outer fabric.

#### Dynamic moisture transfer method

According to the standard GB/T21655.2-2019“Evaluation of moisture absorption and quick drying of textiles-part 2: dynamic moisture transfer method”, using the Q290 liquid moisture management analyzer (standard group (HK) Ltd.) to test the liquid moisture management of fabrics. The MMT is designed to sense, measure and record the liquid moisture transport behavior in multiple directions. The water content in fabric determines the measured electrical resistance. This principle is employed in MMT to obtain moisture management indices for top and bottom layers of fabric. The main parameters of MTT were shown in the following^11^:

Wetting time (WTI –inner surface and WTO –outer surface). It is the time in seconds when the inner and outer surfaces of the specimen begin to be wetted after the test is started.

Absorption rate (ARI – top inner surface and ARO – bottom outer surface). It is the average speed of liquid moisture absorption for the top inner and bottom outer surfaces of the specimen during the initial change of water content during a test.

Maximum wetted radius (MWRI – top inner surface and MWRO – bottom outer surface). It is the greatest ring radius measured on the top and bottom surfaces.

Spreading speed (SSI –inner surface and SSO –outer surface). It is the accumulated rate of surface wetting from the center of the specimen, where the test solution is dropped to the maximum wetted radius.

Accumulative one-way transport capability (AOTI). It is the difference between the area of the liquid moisture content curves of the top and bottom surfaces of the specimen with respect to time.

Overall moisture management capacity (OMMC). It is an index to indicate the overall capability of the fabric to manage the transport of liquid moisture, which includes three aspects of performance: the moisture absorption rate on the bottom side, the one-way liquid capability and the moisture drying speed on the bottom side, which is represented by the accumulative spreading speed.

### Statistical analysis

One-way analysis of variance (ANOVA) is used to compare whether there are significant differences in the means of three or more independent groups. Its core is to calculate the F statistic by decomposing the total variance into between-group variance (treatment effect) and within-group variance (random error). ANOVA was performed at a confidence level of 95% to obtain F and p-values for all the test samples. The strength and direction of association between the different moisture management indices were determined by Pearson correlation coefficient^11^. Pearson correlation coefficient can measure the strength and direction of a linear relationship between two continuous variables, and for absolute value, the closer you get to 1, the stronger the linear independence; 0 means no linear correlation.

## Results and discussion

### Structure and properties of yarns

Nylon multifilament with different fineness and wool short fiber yarns were combined and twisted to form yarns with different fineness and wool content. Table 3 showed the structural parameters of wool fiber and moisture absorption and perspiration nylon ply yarn. It can be seen from the table that the content of wool fibers in the numbered 1 # to 9 # ply yarn continues to decline, from the highest 91.9–44.5%; The diameter calculation formula was shown in formula (1) according to textbook of Textile materials:

1 $$d=\frac{{1.1284}}{{\sqrt {0.75Nm} }}$$

Where the density is 0.75 g / cm^3^, Nm is the metric count. The variation range of plied yarn diameter is 0.16 ~ 0.23 mm. It can be seen that in the same series of 1 ~ 3,4 ~ 6 and 7 ~ 9, the yarn fineness range changes little, which is 0.02 mm.

**Table 3:** Structure parameters of wool fiber and nylon ply yarn.

Combined twist ply yarn	Metric count of wool yarn	Number of wool yarn	Nylon multifilament	Number of Nylon multifilament	wool content of ply yarn (%)	Nylon content of ply yarn (%)	Metric count of ply yarn	Diameter of ply yarn (mm)
1^#^	80	2	20D/24f	1	91.9	8.1	37	0.21
2^#^	80	2	40D/36f	1	85	15	34	0.22
3^#^	80	2	70D/68f	1	76.2	23.8	31	0.23
4^#^	80	1	20D/24f	1	85	15	68	0.16
5^#^	80	1	40D/36f	1	74	26	59	0.17
6^#^	80	1	70D/68f	1	61.6	38.4	49	0.19
7^#^	80	1	20D/24f	2	74	26	37	0.21
8^#^	80	1	40D/36f	2	58.7	41.3	34	0.22
9^#^	80	1	70D/68f	2	44.5	55.5	31	0.23

**Fig. 3:**
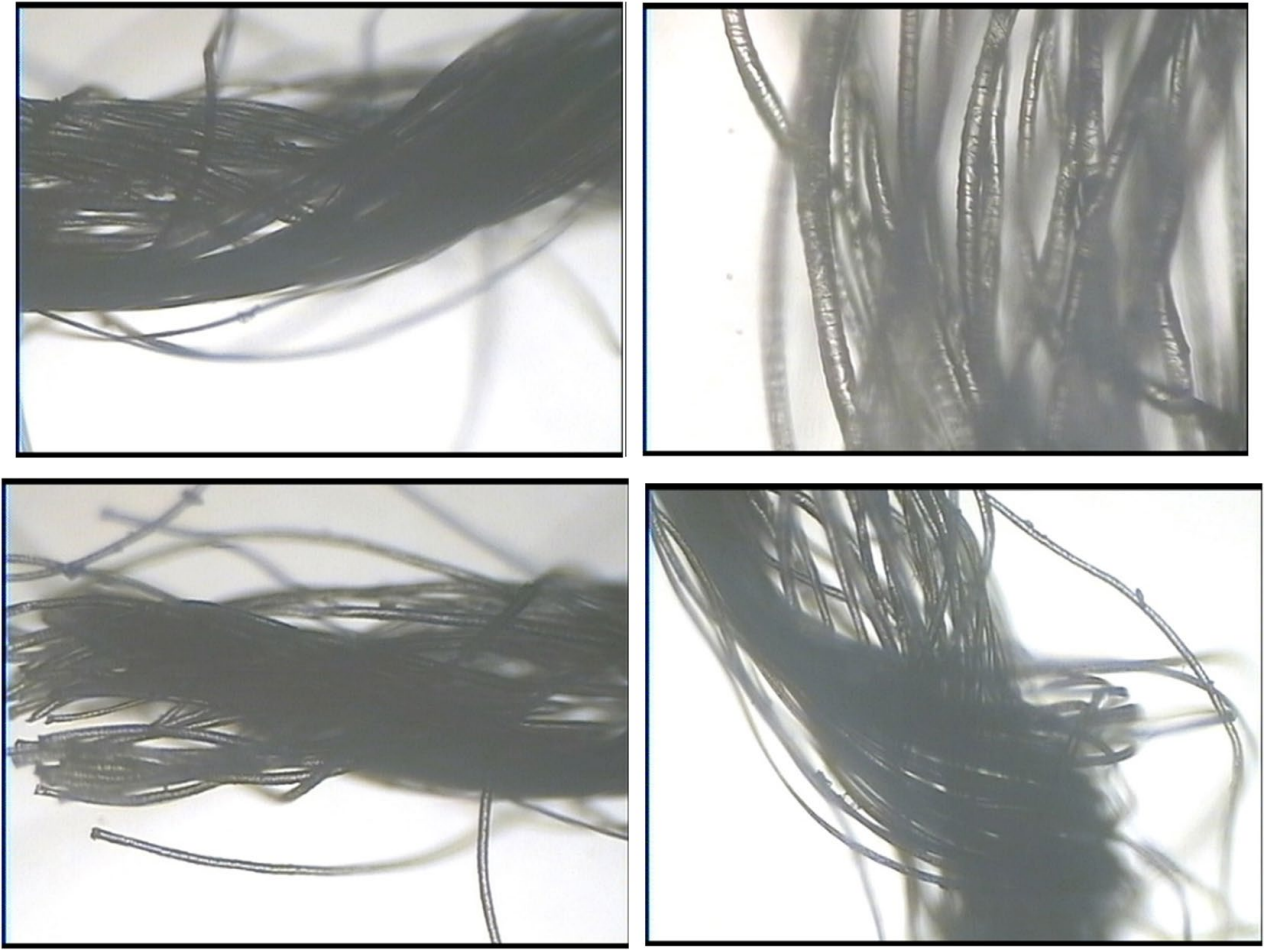
Photos of 1# yarn under optical microscope (4 × 10).

**Fig. 4:**
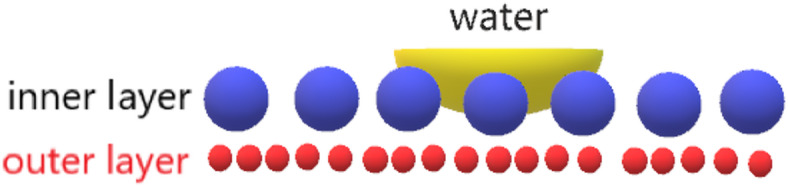
Schematic diagram of water transfer due to the differential capillary effect.

Figure 3 showed photos of the yarn under an optical microscope. As can be seen from the picture, the wool and nylon filaments are twisted together. 20 μm wool fiber was covered with a layer of scales, which made the surface of wool fiber have a certain water repellent effect. Nylon multifilament is also made up of many smaller monofilaments, 70D/68F nylon monofilaments having a fineness of about 2 μm and 20D/24F and 20D/234F monofilaments having a fineness of about 3 μm. The differential capillary effect is formed by the gap between the monofilaments and the fineness of the monofilament, which results in the good moisture absorption and perspiration performance of the nylon multifilament. At the same time because of wool fibers in 15–16% moisture regain, and nylon filament in 4.5%. There are differences in hygroscopicity and hydrophilicity between the two, and nylon filaments are thinner, which are easy to conduct moisture in wool fibers and form a good conduction pathway for sweat, the fabric can be designed as a double-sided fabric with differential capillary effect and unidirectional moisture transfer. The schematic diagram of water transfer due to the differential capillary effect was shown in Fig. 4. Because the inner yarn has a large diameter, and the outer yarn is fine and has a small diameter, the differential capillary effect can be generated, so that the moisture produced by human sweat can be transmitted to the outer layer through the wicking effect.

### Structure parameters of fabrics

Table 4 showed the structural design parameters of the unidirectional moisture-transfer double-layer fabric. Differences of wool content were calculated by the wool content of outer layer yarn minus the wool content of inner layer yarn; Difference of metric number is the metric number of outer layer yarn minus that of inner layer yarn.

It can be seen from the table that there are not only differences in wool content between the inner and outer yarns, but also differences in fineness. It is hoped that through the differential effect of these two aspects, the unidirectional moisture-transfer function can be realized. It can be seen from the table that 1/9 fabric has the highest difference in wool content, which is 47.40%, and 5/7 inner and outer wool content is the same, and the difference is 0. Due to the small difference in the diameter of the plied yarn and the inaccuracy of using the diameter to represent the fineness, the difference in the number of metric branches is used to represent the change in yarn fineness. The fineness difference between the inner and outer strands ranges from-28 to 28.

**Table 4:** Structure design parameters of unidirectional moisture transfer double-layer fabric.

Fabric sample	Yarns for outer layer				Yarns for inner layer				Difference for outer and inner layer	
	Yarn sample	Wool content	Nylon content	Metric number	Yarn sample	Wool content	Nylon content	Metric number	Difference of wool content (%)	Difference of metric number
1/9	1	91.9	8.1	37	9	44.5	55.5	31	47.4	6
1/8	1	91.9	8.1	37	8	58.7	41.3	34	33.2	3
1/6	1	91.9	8.1	37	6	61.6	38.4	49	30.3	-12
1/7	1	91.9	8.1	37	7	74	26	37	17.9	0
1/5	1	91.9	8.1	37	5	74	26	59	17.9	-22
1/3	1	91.9	8.1	37	3	76.2	23.8	31	15.7	6
1/2	1	91.9	8.1	37	2	85	15	34	6.9	3
2/9	2	85	15	34	9	44.5	55.5	31	40.6	9
2/8	2	85	15	34	8	58.7	41.3	34	26.3	3
2/6	2	85	15	34	6	61.6	38.4	49	23.5	0
2/7	2	85	15	34	7	74	26	37	11.1	-15
2/5	2	85	15	34	4	74	26	59	11.1	-3
2/3	2	85	15	34	3	76.2	23.8	31	8.8	-25
3/9	3	76.2	23.8	31	9	44.5	55.5	31	31.7	3
3/8	3	76.2	23.8	31	8	58.7	41.3	34	17.5	0
3/6	3	76.2	23.8	31	6	61.6	38.4	49	14.6	-3
3/7	3	76.2	23.8	31	7	74	26	37	2.3	-18
3/5	3	76.2	23.8	31	5	74	26	59	2.3	-6
4/5	4	85	15	68	5	74	26	59	11.1	-28
5/9	5	74	26	59	9	44.5	55.5	31	29.5	28
5/8	5	74	26	59	8	58.7	41.3	34	15.3	25
5/6	5	74	26	59	6	61.6	38.4	49	12.4	10
5/7	5	74	26	59	7	74	26	37	0	22
7/9	7	74	26	37	9	44.5	55.5	31	29.5	6
7/8	7	74	26	37	8	58.7	41.3	34	15.3	3
7/6	7	74	26	37	6	61.6	38.4	49	12.4	-12
6/9	6	61.6	38.4	49	9	44.5	55.5	31	17.1	18
6/8	6	61.6	38.4	49	8	58.7	41.3	34	2.9	15
8/9	8	58.7	41.3	34	9	44.5	55.5	31	14.2	3

**Fig. 5:**
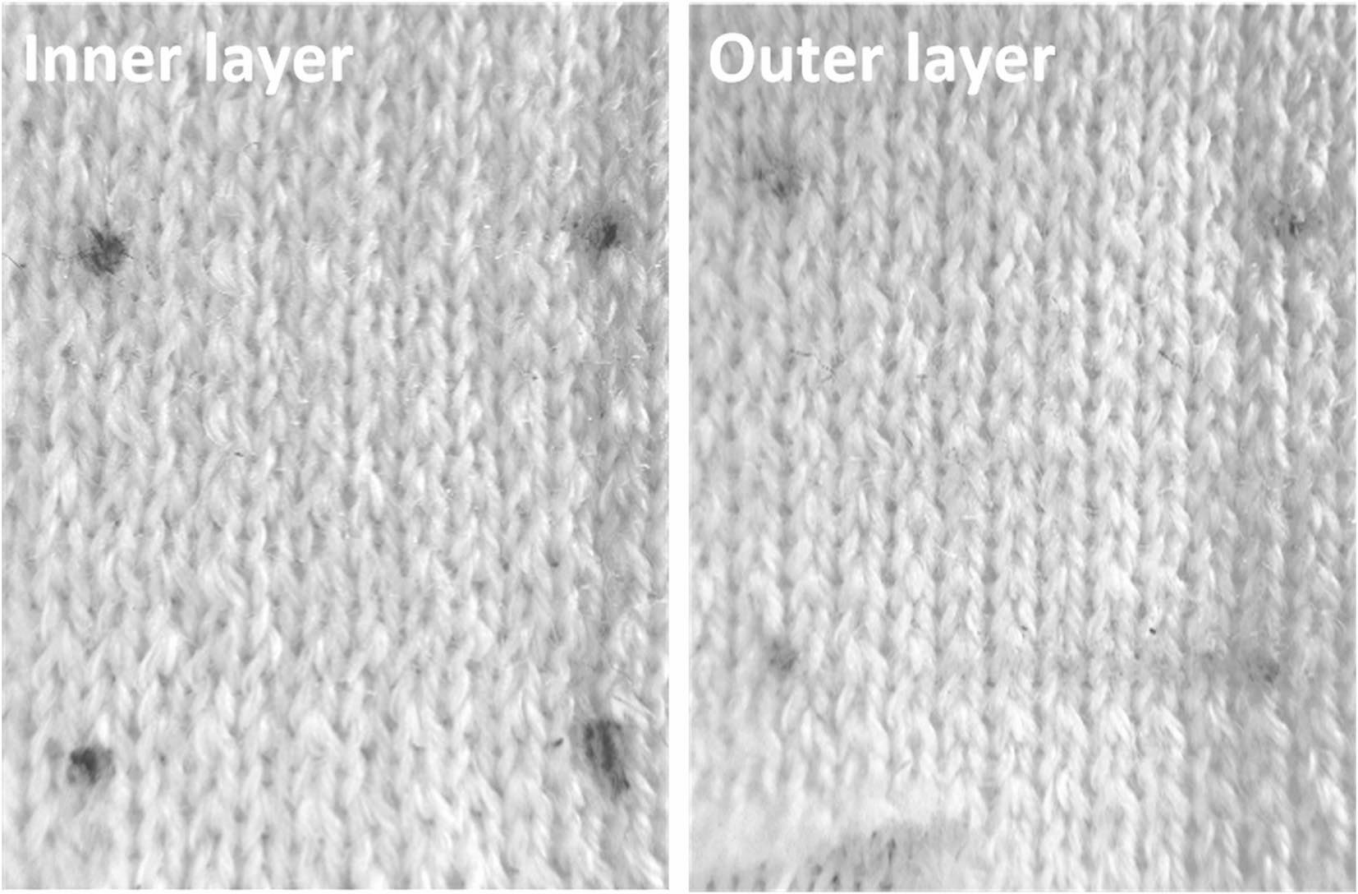
Photos of the inner and outer layer of the fabric.

Figure 5 showed the photos of the front and back sides of the fabric. It can be seen from the figure that the horizontal density of the front and back sides of the fabric is 10 vertical lines per cm, and the vertical density is 14 horizontal lines per cm. The texture and density of the two-sided fabric are exactly the same.

### Contact angle

**Fig. 6:**
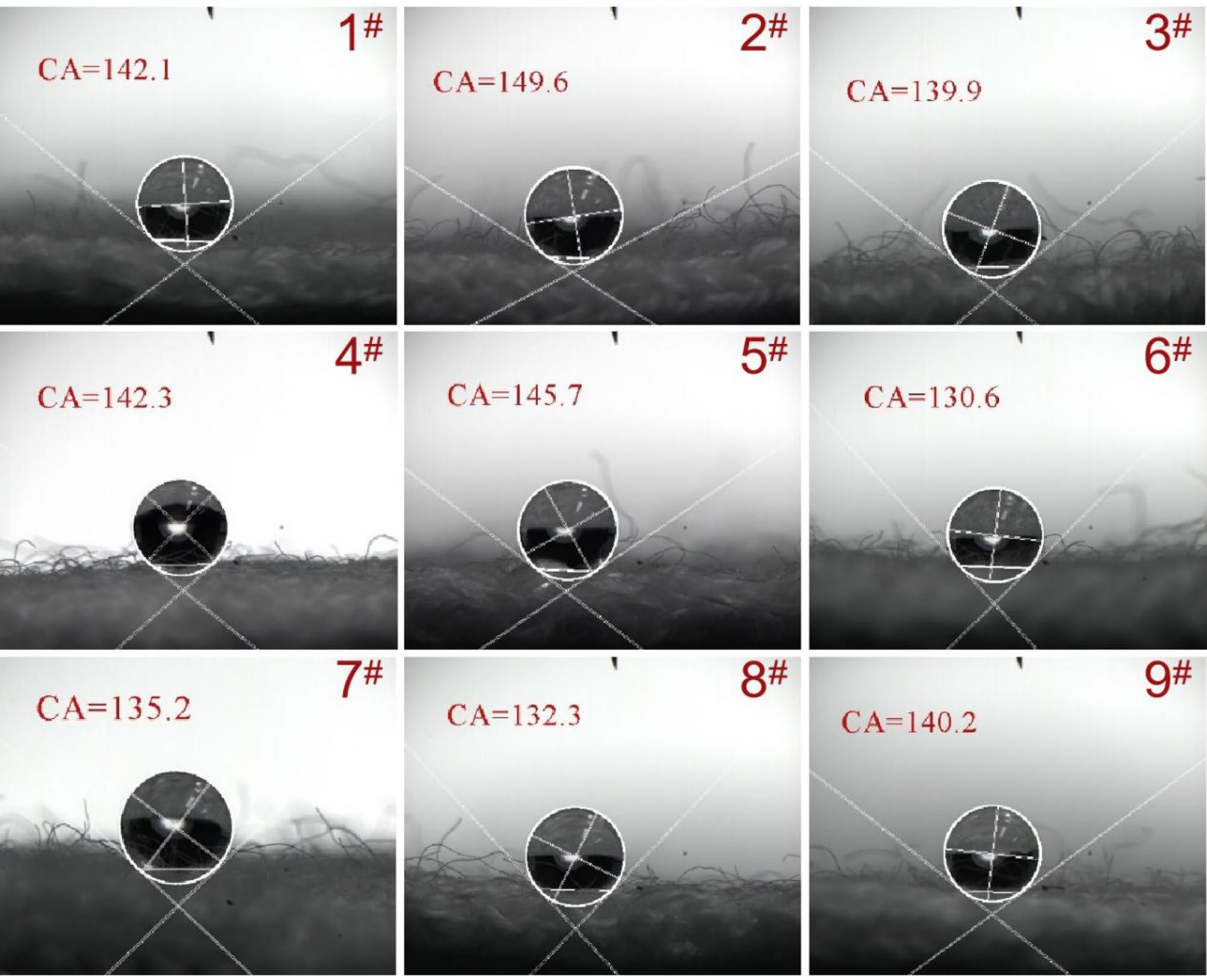
Contact angle of Fabrics made by No. 1–9 # yarns.

Figure 6 showed the contact angles of the fabrics prepared by 1 # and 9 # yarns. It can be seen that all fabrics show excellent hydrophobic properties. The instantaneous contact angle of water is all above 130 °, and the contact angle of the fabric knitted by 2 # yarn even reaches 149.60 °. Due to the presence of wool scales and the hydrophobic surface of nylon, the surface of the fabric is hydrophobic. However, this hydrophobicity presents a non-equilibrium infiltration of fibers due to the hygroscopicity and multiple fineness differences of wool, that is, after the liquid contacts with the fiber, its state will change after a certain period of time. For non-equilibrium infiltration, it is a spreading process, which has been theoretically transformed into an adsorption process of hydrogen bonding or chemical bonding. Therefore, it is necessary to systematically test the moisture management ability of fabrics.

### AOTI

Through the liquid water management tester MMT measurement, the liquid humidity transfer state and the three-dimensional direction on the fabric can be displayed, reflecting the human perception of comfort and humidity. Table 5 showed the MMT test index of the liquid water management tester. AOTI refers to the ability of liquid water to transfer from the water surface of the fabric to the permeable surface, which is expressed as the ratio of the difference between the water absorption on both sides of the fabric to the test time. The unidirectional moisture transfer performance can be evaluated by the cumulative unidirectional transfer capability. It can be seen from Table 5 that AOTIs of 1/9, 1/8, 1/6, 1/7 and 1/5 are more than 400, and according to the GB/T21655.2-2019, the rating is 5, which has excellent one-way moisture capacity. AOTIs of 7/9, 7/8 and 7/6 samples are all above 300, and the rating is also 5, which also has excellent one-way moisture conductivity. The rating is 4 when the AOTI is among 200 ~ 300, and the one-way moisture transfer performance is very good.

Considering comprehensively, the surface and inner layer moisture soaking time, water absorption rate, wetting radius, liquid moisture diffusion rate, cumulative one-way moisture conductivity index and OMMC these indicators, 1/7,7/8 can be rated as water seepage samples; 1/3, 1/2, 2/3, 2/9, 2/8, 2/7, 2/6, 2/5, 3/6, 6/9 could be evaluated as hygroscopic and sweat-relieving water management samples. 1/9, 1/8, 1/6, 1/5, 3/5, 3 /9, 3/8, 3/7, 5/9, 5/8, 5/7, 5/6, 6/8, 7/9, 8/9, 7/6 can be evaluated as water management samples. 4/5 can be evaluated as fast absorption and slow drying. The water seepage sample means that the fabric has a small diffusion area and very good one-way transmission performance; moisture management samples refer to the moisture in the fabric can be medium to fast absorption, medium to fast diffusion, large area diffusion of the lower surface, fast diffusion of the lower surface, good to excellent unidirectional transmission. Fast absorption and slow drying refer to the medium to fast absorption, medium to fast slow diffusion, small diffusion area and slight one-way transmission of water in the fabric.

**Table 5:** MMT test index.

Sample	Difference of wool content	Difference of linear density	WTI (s)	WTO (s)	ARI (%/sec)	ARO (%/sec)	MWRI (mm)	MWRO (mm)	SSi T (mm/sec)	SSO (mm/sec)	AOTI	OMMC
1/9	47.40	6	22.20	9.19	8.35	19.17	11.67	16.67	0.45	0.91	485.13	0.53
1/8	33.20	3	36.26	6.86	9.65	21.71	10.00	13.75	0.32	0.91	490.07	0.53
1/6	30.30	-12	16.64	10.20	17.38	51.80	11.25	15.00	0.61	0.83	412.01	0.54
1/7	17.90	0	20.57	8.68	10.90	23.43	8.75	8.75	0.61	0.85	446.47	0.50
1/5	17.90	-22	23.94	7.66	13.76	21.20	12.50	15.00	0.55	0.97	429.56	0.51
1/3	15.70	6	5.88	18.05	18.55	52.69	10.00	13.75	0.91	0.52	262.89	0.47
1/2	6.90	3	6.91	19.32	27.58	71.65	10.00	15.00	0.82	0.50	300.55	0.56
2/9	40.60	9	15.59	11.18	34.35	51.54	13.75	15.00	0.81	0.94	282.80	0.49
2/8	26.30	3	21.75	7.34	16.38	52.14	12.50	15.00	0.60	1.01	254.49	0.46
2/6	23.50	0	16.69	9.29	16.87	43.37	13.75	15.00	0.76	0.92	270.04	0.45
2/7	11.10	-15	12.45	13.22	13.05	43.97	15.00	16.25	0.89	0.75	208.92	0.38
2/5	11.10	-3	19.71	7.39	19.97	37.37	15.00	17.50	0.78	1.19	290.77	0.47
2/3	8.80	-25	12.71	15.89	46.54	92.48	10.00	12.50	0.72	0.57	183.69	0.45
3/9	31.70	3	18.81	14.96	20.88	34.73	10.00	11.25	0.69	0.64	238.58	0.39
3/8	17.50	0	5.98	16.25	16.16	65.34	10.00	11.25	0.93	0.47	161.72	0.39
3/6	14.60	-3	11.35	13.95	25.51	29.20	11.25	13.75	0.77	0.76	244.14	0.38
3/7	2.30	-18	20.09	15.87	18.22	20.61	11.25	12.50	0.68	0.63	162.44	0.27
3/5	2.30	-6	17.29	9.36	13.49	20.40	11.25	15.00	0.61	0.88	268.81	0.38
4/5	11.10	-28	9.69	14.22	20.08	53.35	15.00	13.33	1.04	0.68	86.91	0.27
5/9	29.50	28	8.91	10.20	15.05	28.09	15.00	17.50	1.14	1.04	220.26	0.36
5/8	15.30	25	10.36	10.55	16.30	26.89	15.00	18.75	1.02	1.03	168.97	0.30
5/6	12.40	10	10.62	9.63	17.27	30.86	16.25	20.00	1.10	1.17	212.55	0.36
5/7	0.00	22	16.87	7.93	13.69	17.20	15.00	15.00	0.80	0.93	246.83	0.35
7/9	29.50	6	15.52	11.04	10.49	22.91	11.25	16.25	0.69	0.93	392.15	0.49
7/8	15.30	3	26.12	6.29	10.10	14.43	13.75	12.50	0.44	0.99	457.47	0.52
7/6	12.40	-12	25.20	9.01	12.53	17.40	13.75	15.00	0.61	0.94	363.06	0.46
6/9	17.10	18	12.27	10.18	13.64	32.92	13.75	15.00	0.84	0.94	242.27	0.40
6/8	2.90	15	14.78	10.74	14.31	22.13	16.25	17.50	0.97	0.98	221.67	0.34
8/9	14.20	3	11.16	11.49	13.48	47.71	11.25	13.75	0.90	0.83	212.15	0.40

**Fig. 7:**
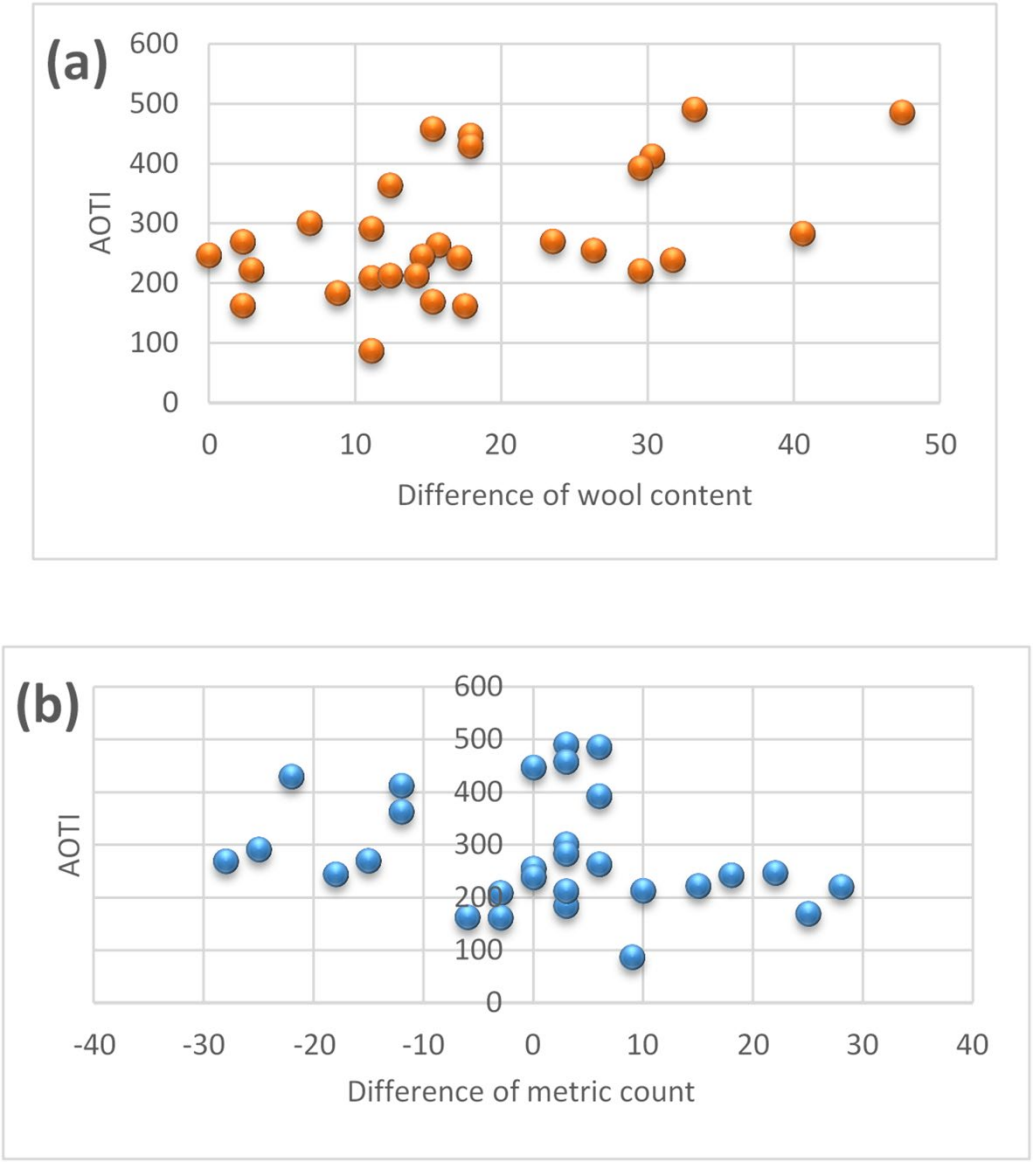
The relationship between the difference between wool content and metric count and AOTI.

Figure 7 showed the scatter plot between AOTI and the difference in wool content and metric count. It can be seen from the figure that the difference of wool content roughly has a positive correlation with AOTI. The maximum cumulative one-way moisture capacity is obtained when the wool content difference is the highest, while the fineness difference does not show a significant correlation effect. On the contrary, when the fineness difference is not large (among 0 ~ 10), the cumulative one-way moisture capacity is larger. The ANOVA significant effect analysis and Pearson correlation analysis were used to analyze the data. The results were shown in Table 6.

**Table 6:** ANOVA significant effect analysis and pearson correlation analysis.

Parameters	Analyze	Difference of wool content	Difference of metric count
WTI (s)	Pearson correlation	0.275	-0.31
	Significance ( two-tailed)	0.149	0.102
WTO (s)	Pearson correlation	-0.222	0.074
	Significance (two-tailed)	0.246	0.703
ARI (%/sec)	Pearson correlation	-0.099	-0.059
	Significance (two-tailed)	0.609	0.76
ARO (%/sec)	Pearson correlation	-0.207	0.303
	Significance (two-tailed)	0.281	0.11
MWRI (mm)	Pearson correlation	-0.027	-0.015
	Significance (two-tailed)	0.889	0.939
MWRO (mm)	Pearson correlation	0.018	0.226
	Significance (two-tailed)	0.925	0.238
SSI(mm/sec)	Pearson correlation	-0.316	0.465*
	Significance (two-tailed)	0.095	0.011
SSO(mm/sec)	Pearson correlation	0.164	0.074
	Significance (two-tailed)	0.395	0.703
AOTI	Pearson correlation	0.482**	-0.247
	Significance (two-tailed)	0.008	0.196
OMMC	Pearson correlation	0.491**	-0.314
	Significance (two-tailed)	0.007	0.097

### Effect of wool content difference

Table 7 showed the result of one-way ANOVA for wool content difference. It can be seen from the table that the difference of wool content has a significant effect on AOTI (*p* = 0.008 < 0.05). The difference of high wool content plays a positive role in the infiltration and transmission of water. It also proves the possibility of designing high-content wool one-way moisture-conducting fabric.

**Table 7:** Result of one-way ANOVA for wool content difference effecting on AOTI.

	Sum of squares	Degrees of freedom	Mean square	F value	*p* value
Regression	74090.603	1	74090.603	8.152	0.008
Residual	245381.404	27	9088.2		
Total	319472.00	28			

It can also be seen from Table 6 that the difference in wool content has a significant effect on OMMC (*p* = 0.007 < 0.05), and the Pearson correlation test also shows a significant correlation between the difference in wool content and AOTI and OMMC. However, the difference of wool content has no significant correlation and influence on other indexes such as wetting time and diffusion rate of water.

In the process of unidirectional liquid transport, the liquid management in the fabric includes three steps : conduction, absorption and evaporation. As shown in Fig. 2 of the unique structure of the formed knits, when the liquid is initially in contact with the hydrophobic transport layer with less wool content in the inner layer, the rising capillary force (F) spontaneously pumps the liquid from the inner layer to the outside. This capillary force is sufficient to overcome viscous resistance (Fv) and self-gravity (G). More importantly, the capillary force comes from the capillary pressure (P), and the difference in capillary radius and hygroscopicity leads to the capillary pressure difference (ΔP) at the interface. The action of capillary pressure makes the liquid move rapidly in an open space. Capillary flow in liquids is driven by a combination of the cohesive force inside the liquid itself and the adhesion force between the liquid and the solid surface^7^.

Therefore, based on the structure of wool / nylon double-sided weft plain knitted fabric, the unidirectional wetting behavior of the fabric is first explained by the capillary channel model. Here, the inner nylon monofilament is used as the hydrophobic layer, the inner wool fiber and the middle layer yarn are used as the transfer layer, and the outer nylon monofilament and wool fiber are used as the evaporation layer. When the water droplet initially falls on the hydrophobic side of the inner layer, it maintains the Wenzel-Cassie state and is subjected to the dual effects of hydrophobic force and water droplet surface tension (f_1_). Hydrophobicity is related to the osmotic pressure of the hydrophobic layer. When the surface tension of the droplet is greater than the hydrophobic HF, the droplet enters the capillary channel between the fibers and is affected by the Laplace pressure. Here, the size of the Laplace pressure is given by the Yang-Laplace equation^4^.

2 $${\text{P}}=({\text{4}}\gamma {\text{cos}}\theta)/{\text{D}}$$

Where P is the Laplace pressure, γ is the liquid-solid interfacial tension, θ is the contact angle, and D is the pore size of the fiber. Due to the different contact angles and pore sizes of the hydrophobic layer, the transfer layer and the evaporation layer, the Laplace pressure of the droplets in the capillary channel is also different. When the droplet is at the interface between the hydrophobic layer and the transfer layer, the droplet is subjected to the Laplace forces P_1_ and P_2_ in the vertical direction, and the resultant force ΔP_1_ is downward.

3 $$\Delta {{\text{P}}_{\text{1}}}\,=\,{{\text{P}}_{\text{1}}} - {{\text{P}}_{\text{2}}}=({\text{4}}\gamma {\text{cos}}{\theta _{\text{1}}})/{{\text{D}}_{\text{1}}} - ({\text{4}}\gamma {\text{cos}}{\theta _{\text{2}}})/{{\text{D}}_{\text{2}}}$$

Because wool fiber has higher hydrophilicity than nylon fiber, water droplets can quickly enter the interior of wool fiber. At the same time, the capillary force between the fibers in the horizontal direction promotes the expansion of water. Similarly, when some droplets enter the interface between the transport layer and the evaporation layer, the droplets are also subjected to the Laplace force in the vertical direction.

According to the characteristics of wettability, due to the high content of outer wool, the moisture content of the outer surface of the fabric is always higher than that of the inner fabric, indicating that once the water contacts the inner fabric, the water can be effectively pushed into the pores by hydrostatic pressure and pulled out by the capillary force of the wool. When the water droplets reach the outer fabric, the water droplets can continuously penetrate the two-layer wetting gradient structure, and finally evaporate in the evaporation layer without reverse wetting.

### Difference of metric count

**Fig. 8:**
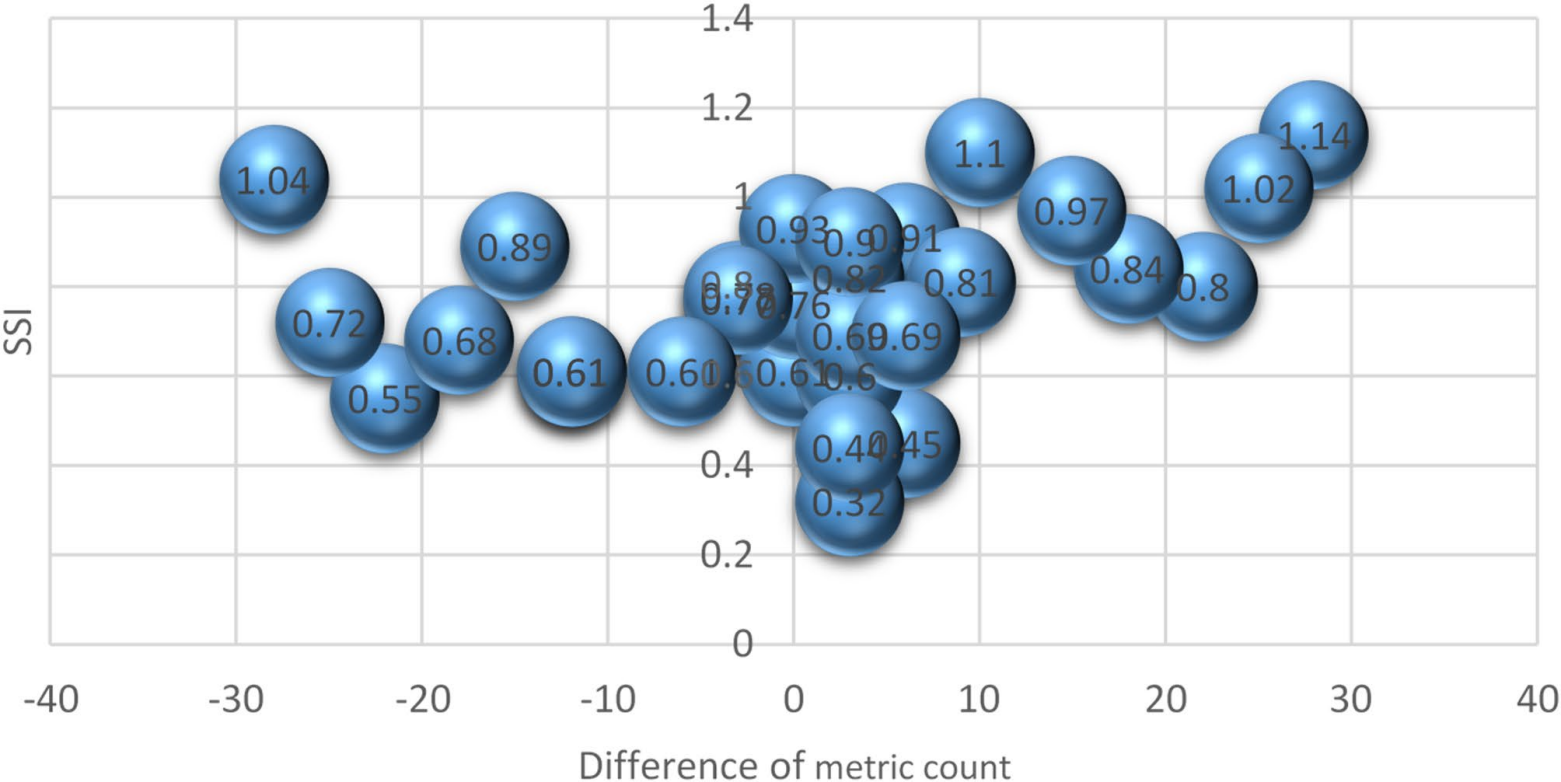
Effect of difference of metric count on SSI.

Table 8 showed the result of one-way ANOVA for difference of metric count effecting on AOTI. From Table 7, it can be seen that the difference in metric count has no significant effect on the AOTI and OMMC, which may be due to the small difference in fineness between the plied yarns. However, it can be seen fromTable 5 that the fineness difference has a significant effect on the surface liquid water diffusion rate (*p* = 0.011 < 0.05) and a significant correlation (at the 0.5 level), indicating that the fineness difference has a significant effect on the surface water diffusion rate. The greater the fineness difference, the water diffusion rate shows an upward trend (Fig. 8).

**Table 8:** The result of one-way ANOVA for difference of metric count effecting on AOTI.

	Sum of squares	Degrees of freedom	Mean square	F value	*p* value
Regression	0.322	1	0.322	2.166	0.153
Residual	4.017	27	0.149		
Total	4.34	28			

## Conclusions

By designing a ply yarn formed by different content of wool and nylon yarn and a double-sided knitted fabric with different wool content in the inner and outer layers, the one-way moisture conductivity of the fabric with high wool content is realized. Under the condition of maintaining the temperature control of wool fiber and the dryness of skin contact, the moisture absorption and perspiration and one-way moisture conductivity of the fabric can be increased, which can be applied to sportswear fabrics. The results also show that the difference in wool content between the inner and outer layers has a significant effect on the cumulative one-way moisture index, while the small change in yarn linear density has no significant effect on the cumulative one-way moisture index, but has a significant effect on the surface diffusion rate. The cumulative one-way moisture conductivity indexes of samples 1 / 9, 1 / 8, 1 / 6, 1 / 7 and 1 / 5 are all above 400, and the rating is 5, which has excellent one-way moisture conductivity.

1 / 7 and 7 / 8 can be evaluated as water seepage samples. In the future, the difference of yarn linear density can be further expanded, and the wicking effect can be increased, so as to further improve the moisture absorption and perspiration and one-way moisture conductivity.

## Data Availability

All data generated or analyzed during this study are included in this published article.
